# SYSBIONS: nested sampling for systems biology

**DOI:** 10.1093/bioinformatics/btu675

**Published:** 2014-10-16

**Authors:** Rob Johnson, Paul Kirk, Michael P. H. Stumpf

**Affiliations:** Centre for Integrative Systems Biology and Bioinformatics, Department of Life Sciences, Imperial College London, London SW7 2AZ, UK

## Abstract

**Motivation:** Model selection is a fundamental part of the scientific process in systems biology. Given a set of competing hypotheses, we routinely wish to choose the one that best explains the observed data. In the Bayesian framework, models are compared via Bayes factors (the ratio of evidences), where a model’s evidence is the support given to the model by the data. A parallel interest is inferring the distribution of the parameters that define a model. Nested sampling is a method for the computation of a model’s evidence and the generation of samples from the posterior parameter distribution.

**Results:** We present a C-based, GPU-accelerated implementation of nested sampling that is designed for biological applications. The algorithm follows a standard routine with optional extensions and additional features. We provide a number of methods for sampling from the prior subject to a likelihood constraint.

**Availability and implementation:** The software SYSBIONS is available from http://www.theosysbio.bio.ic.ac.uk/resources/sysbions/

**Contact:** m.stumpf@imperial.ac.uk, robert.johnson11@imperial.ac.uk

## 1 INTRODUCTION

Given a set of models proposed to explain some observation, we seek to rank them according to the extent to which they are supported by some data. Likelihood-based approaches find the point at which the likelihood function is maximized, and compare models based on these maxima (Burnham and Anderson, 2002). Bayesian approaches for model selection rest on Bayes factors: the ratio of evidences of competing models. A number of methods exist to estimate the evidence (Kirk *et al.*, 2013), a metric of the support afforded to a model by some data.

Nested sampling is a Bayesian method for evidence estimation and parameter inference for systems where a likelihood function can be defined (Skilling, 2006). As the algorithm progresses, it generates samples from the posterior parameter distribution directly.

We present a C-based nested sampling tool for computational biologists. The user supplies a likelihood function, some experimental data and the prior parameter distribution. The program returns a value for the evidence alongside samples from the posterior parameter distribution. There exists a Fortran-based nested sampling package, MultiNest (Feroz *et al.*, 2009), used in the astrophysics community. Our work is aimed specifically at the biological community and includes an SBML (Systems Biology Markup Language, Rodriguez *et al.*, 2007) parser so that models can be specified according to current standards. The recent growing use of nested sampling in systems biology invites the release of a tool implementing the method (Aitken and Akman, 2013; Burkoff *et al.*, 2012; Dybowski *et al.*, 2013; Kirk *et al.*, 2013; Pullen and Morris, 2014).

## 2 APPROACH

The evidence is defined as $Z=\int_{\Theta }\;\ell (\theta )\pi (\theta )\text{ d}\theta$, where θ is the parameter set (and Θ the parameter space), ℓ the likelihood function and π the prior. The change in notation π(θ) dθ=dX(θ), where X(θ) is the cumulative density function, allows the integral to be written $Z=\int_{0}^{1}\;\ell (\theta )\text{ d}X(\theta )$. This can be approximated as a sum, $Z\approx \sum_{i=1}^{N}\ell_{i}W_{i}$, where *N* points are sampled and *W_i_* is the proportion of prior mass represented by point *i*, calculated as the difference between the volume enclosed by the contour of constant likelihood through $\ell_{i}$ and that through $\ell_{i-1}$. Nested sampling is a method for generating the sequence of points $\left\{\ell_{i},W_{i}\right\}$.

For a thorough presentation of nested sampling, we refer the reader to the work of Skilling (2006) and Sivia and Skilling (2006). For our purposes, we follow the general algorithm:

1. Initialise *Z* = 0

2. Generate *N* points from π(θ)

3. **for** i=1:M

 a. Find $\theta^{*}$ with lowest likelihood, $\ell^{*}$

 b. Calculate $W_{i}=\exp\left(-\frac{i-1}{N}\right)-\exp\left(-\frac{i}{N}\right)$

 c. Set $Z=Z+\ell^{*}W_{i}$

 d. Resample $\theta^{*}∼\pi (\theta ){|}_{\ell (\theta )>\ell^{*}}$

4. **end for**

5. Set $Z=Z+\sum_{j=1}^{N}\ell_{j}\exp\left(-\frac{M}{N}\right)/N$

Our program is written primarily in C with additional capability for GPU acceleration. Other features include an SBML parser for automated generation of likelihood functions (Liepe *et al.*, 2010) and plotting tools. For the task of sampling from the prior subject to a likelihood constraint (step 3d), we provide three methods. The accuracy of the approximation in step 3b depends on the population of *N* points (live points) being truly distributed as the prior within the given likelihood constraint (Skilling, 2006).

## 3 METHODS

Our nested sampling package is a command-line tool for Linux and MacOSX platforms. Pre-requisites are listed in the accompanying manual. The user supplies a likelihood function, either by editing a template file or using an SBML file. An executable is then made that receives input from the command line. When the program is run, live points are generated and their likelihoods evaluated according to the function supplied by the user. On completion, it returns the calculated evidence with standard deviation, samples from the posterior, trajectories generated by points from the posterior and files from which the algorithm can be restarted.

### 3.1 Algorithm options

Options available to the user are listed in Table 1. The only required input is the parameter set (all other variables have default values). Parameters may be constant or inferred subject to a uniform prior distribution, for which lower and upper bounds must be supplied. The algorithm can be terminated either by specifying the number of iterations, or by monitoring the rate at which the evidence accumulates: the loop terminates at iteration *m* if $\ell_{m}W_{m}/\sum_{i=1}^{m}\ell_{i}W_{i}<tol$.

**Table 1.** Input options

Variable	Tag	Input	Default
Number of live points	nLive	**integer	1000
Number of iterations	maxIter	**integer	on, 10 000
Tolerance	tol	decimal	off, 0.001
*Parameters	constant	**value	none
	uniform	** bounds	none
Sampling method	rejection	none	off
	rw	none	off
	ellipsoid	expansion factor	on, 2
Restart from file	Restart	file paths	_restart_points.txt,
			_restart_input.txt
Write restart	write_restart	file path root	_restart
Points to leap	nLeap	**integer	1
Adaptive leaping	adaptive	none	off
CUDA	cuda	**nLeap,	off, none,
		**max. threads	none

*required; **required if tag given.

### 3.2 Sampling methods

We include three sampling methods for step 3d of the algorithm: rejection, for perfectly sampling from the prior, and random walk (following Sivia and Skilling, 2006) and ellipsoidal (following Feroz *et al.*, 2009) for refined sampling with reduced computational cost.

*Rejection*: The rejection method samples from the prior as initially defined, accepting the point if its likelihood value is within the constraint and rejecting otherwise. This method remains true to the requirement that samples are taken from the prior subject to the likelihood constraint, but its efficiency is poor: as the lowest likelihood increases, the acceptance rate becomes prohibitively small.

*Random walk*: The random-walk method duplicates a point randomly chosen from the current live-point population and walks it 20 steps, accepting the new point at each step if its likelihood is within the constraint. The steps are scaled according to the covariance among the present population, and scaled further to converge to an acceptance rate of 0.5 (Sivia and Skilling, 2006).

*Ellipsoidal*: The ellipsoidal method (Mukherjee *et al.*, 2006) creates an ellipsoid surrounding the current population of live points, expanded by some user-supplied factor. The new point is sampled from within the ellipsoid. This increases the acceptance rate but risks excluding areas of prior mass that lie inside the current likelihood constraint.

### 3.3 Output

A summary file of input and output information is created, documenting the number of live points, number of iterations, tolerance, sampling method and parameter ranges, followed by the evidence with standard deviation, the prior-to-posterior information gain and the means of all parameters and their standard deviations. Posterior distributions of the parameters can be plotted individually as histograms and in pair-wise scatter plots using the data stored in the posterior file. Finally, a file of trajectories is created that can be compared against the input data.

Restart files are created, documenting input parameters that must persist upon restart (such as the number of live points) and listing all points, live and discarded. These files can be used to restart the program from where it completed. It is also possible to specify the path to where the restart files are written.

## 4 SUMMARY

We present SYSBIONS, a computational tool for model selection and parameter inference using nested sampling. Using a data-based likelihood function, our package calculates the evidence of a model and the corresponding posterior parameter distribution.
